# Histopathological Investigation of Dura-like Membrane in Vestibular Schwannomas

**DOI:** 10.3390/brainsci11121649

**Published:** 2021-12-15

**Authors:** Yumiko Oishi, Ryota Tamura, Kazunari Yoshida, Masahiro Toda

**Affiliations:** Department of Neurosurgery, Keio University School of Medicine, Tokyo 160-8582, Japan; ysyosk2010028@gmail.com (Y.O.); moltobello-r-610@keio.jp (R.T.); kazrmky@keio.jp (K.Y.)

**Keywords:** vestibular schwannoma, dura, membrane, arteriogenesis, angiogenesis, αSMA

## Abstract

The dura-like membrane (DLM) is an outermost membranous structure arising from the dura mater adjacent to the internal auditory meatus (IAM) that envelops some vestibular schwannomas (VSs). Its recognition is important for the preservation of the facial and cochlear nerves during tumor resection. This study analyzes the histopathological characteristics of the DLM. The expression of CD34 and αSMA was histopathologically analyzed in tumor and DLM tissue of 10 primary VSs with and without a DLM. Tumor volume, resection volume percentage, microvessel density (MVD), and vessel diameter were analyzed. Volumetric analysis revealed that the presence of a DLM was significantly associated with lower tumor resection volume (*p* < 0.05). Intratumoral vessel diameter was significantly larger in the DLM group than the non-DLM group (*p* < 0.01). Larger VSs showed a higher intratumoral MVD in the DLM group (*p* < 0.05). Multilayered αSMA-positive vessels were identified in the DLM, tumor, and border; there tended to be more of these vessels within the tumor in the DLM group compared to the non-DLM group (*p* = 0.08). These arteriogenic characteristics suggest that the DLM is formed as the tumor induces feeding vessels from the dura mater around the IAM.

## 1. Introduction

Vestibular schwannomas (VSs) are benign tumors arising from the nerve sheath of the vestibular nerve. Functional preservation of the facial and cochlear nerves during their resection is a key concern [1,2]. Understanding the membranous structure of VSs is important in order to define the ideal surgical plane and perform safe and efficient resection [3].

The dura-like membrane (DLM) is an outermost membranous structure arising from the dura mater adjacent to the internal auditory meatus (IAM) that contains dilated vessels and envelops some VSs [4]. The relationship between DLM and surrounding structures, including the IAM and dura mater, was described in our previous paper [4]. In this study, we demonstrated that recognition of the DLM is important in order to preserve the facial and cochlear nerves during surgical resection of VSs. However, the histopathological features of the DLM have not been fully elucidated and the factors associated with its neogenesis are unknown.

Tumors are supplied by various types of vessels that arise from both arteriogenesis and angiogenesis [5]. Arteriogenesis gives rise to feeder arteries, which are surrounded by vascular smooth muscle cells [6]. Angiogenesis is the formation of granulation tissue and collagen and is stimulated by fibroblast growth factor and blood vessel growth [7].

This study examined arteriogenic factors in the DLM of VSs and revealed the origin of the DLM. The DLM has particular arteriogenic characteristics that may be associated with perioperative clinical course.

## 2. Materials and Methods

### 2.1. Ethical Statement

All procedures performed in this study involving human subjects were in accordance with the ethical standards of the Institutional Ethics Committee (Reference number: 20050002) and with the 1964 Helsinki declaration and its later amendments or comparable ethical standards. Informed consent was obtained from all patients.

### 2.2. Study Population

Ten primary VSs resected via the lateral suboccipital (retrosigmoid) approach between 2014 and 2019 were retrospectively reviewed. Mass reduction was performed in all cases. Neurofibromatosis type 2 VSs were excluded. Cases 1–3 have been previously reported [4]. Tumors were classified according to presence (DLM group) or absence (non-DLM group) of the DLM based on intraoperative findings. The DLM was defined as an outermost thick enveloping membrane containing dilated vessels that appeared to be continuous with the dura mater of the posterior wall of the IAM, as previously described [4]. Gadolinium-enhanced T1-weighted or plane magnetic resonance imaging was used to evaluate tumor volume. Tumor volume was measured using the segmentation method, as previously described [8]. Data regarding extent of resection and patient clinical course were also reviewed.

### 2.3. Immunohistochemical Analysis

A continuous sheet of the DLM was taken from the dura mater on the petrous bone just behind the IAM to the tumor surface and sectioned, as previously reported [4]. Histopathological analyses were performed on 3 μm sections of formalin-fixed paraffin-embedded sections. Immunohistochemistry was performed according to standard procedures, as previously described [8,9]. Masson’s trichrome (MT) staining was also conducted according to standard procedures [10]. Samples were stained with the following primary antibodies: CD34 (1:100; mouse IgG; F1604 Nichirei Biosciences Inc., Tsukiji Chuo-Ku, Japan), anti- α-smooth muscle actin (αSMA) (1:200; mouse IgG; MA1-06110 Thermo Fisher Scientific, Walton, MA, USA), and S-100 (1:2; rabbit IgG; IR504 DAKO). Hematoxylin and eosin, CD34, and αSMA stains were used in all cases. The tumor attached to the DLM was totally removed in cases 1 and 2, which underwent additional staining with S-100. To assess microvessel density (MVD) and vessel diameter, tissue sections were screened using CD34 immunohistochemistry in low-power fields and the three most vascularized regions (hot spots) were selected for automatic microvessel counting under high-power magnification microscopy (0.95 mm^2^) (Biorevo BZ-9000, KEYENCE, Osaka, Japan), as previously described [8]. As vascular smooth muscle cells are the primary component of arterial and venous walls, the vessel wall was assessed based on αSMA expression. Multilayered vessels with positive αSMA expression were defined as arteriogenic vessels.

### 2.4. Statistical Analysis

The Student’s t-test was used to compare tumor volume, tumor resection volume percentage, MVD, and vessel diameter between the DLM group and non-DLM group. Fisher’s exact test was used to compare the presence of multilayered αSMA-positive(+) vessels between the DLM group and non-DLM group. The correlation between MVD and tumor volume in the DLM group was analyzed using the Pearson correlation test. Statistical analyses were performed using SPSS software (IBM Corp., Armonk, NY, USA). *p* < 0.05 was considered significant.

## 3. Results

### 3.1. Patient Characteristics

**Figure 1:**
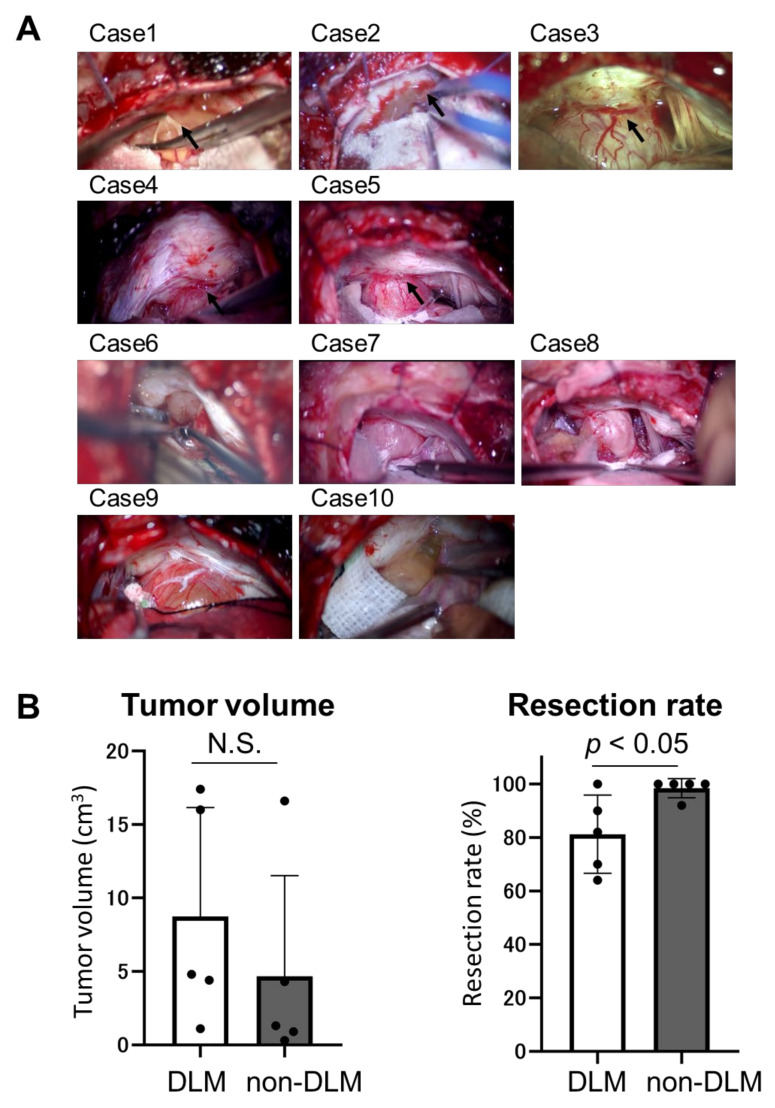
Tumor characteristics. DLM, dura-like membrane. (**A**) Tumors with and without dura-like membrane (DLM). DLM (black arrow) is observed in Cases 1–5, and not observed in Cases 6–10. DLMs contain the dense dilated vessels from the originated dura mater. These vessels are continuously observed on the surface of the tumor. In non-DLM group, these dilated vessels were not noticeable in the dura mater around the internal auditory meatus (IAM). (**B**) Tumor volume and resection rate between DLM and non-DLM group. The average tumor volumes of DLM group and non-DLM group are 8.7 cm^3^ and 4.7 cm^3^, respectively (*p* = 0.39). The resection rate of the DLM group is significantly lower than that in non-DLM group (81.2% and 98.4%, *p* < 0.05). N.S.: not significant.

Patient characteristics are summarized in Table 1. DLM was defined by the intraoperative findings (Figure 1A). Cases 1–5 were classified as the DLM group, and cases 6–10 were classified as the non-DLM group. All cases had a preoperative hearing disorder. Facial nerve palsy was observed in one case of the non-DLM group. No case experienced a recurrence. Average tumor volume in the DLM and non-DLM groups was 8.7 cm^3^ (range, 1.1–17.4) and 4.7 cm^3^ (range, 0.3–16.6), respectively. The difference was not significant (*p* = 0.39) (Figure 1B). There was no cystic tumor in the DLM group; in the non-DLM group, tumors with volume ≥4.0 cm^3^ (cases 9 and 10) contained cystic components.

### 3.2. Intraoperative Findings and Surgical Outcome

Intraoperative findings demonstrated that the DLM enveloped the tumor around the IAM and contained dense dilated vessels arising from the adjacent dura mater (Figure 1A). These vessels continued along the tumor surface. There was a tight adhesion between the DLM and the tumor or nerves, which made preservation of the nerves more challenging at the beginning of the subcapsular dissection. It was difficult to identify correct layer for dissection when the DLM envelopes the tumor and nerves. In the non-DLM group, these dilated vessels were not noticeable in the dura mater around the IAM. Postoperative facial nerve palsy occurred in one case of the DLM group (case 1, permanent) and two of the non-DLM group (cases 6 and 9, temporary). In case 1, the facial nerve was injured during dissection of the tumor capsule, which was tightly attached to its DLM. Although the facial electromyography monitoring was normal throughout the operation in case 6, postoperative facial palsy occurred. In case 9, the facial nerve was injured during exfoliation of the tumor from the brain stem. The resection volume percentage was significantly lower in the DLM group than the non-DLM group (81.2% vs. 98.4%, *p* < 0.05) (Figure 1B). In these patients with DLM, tumor remnants were mainly found around the IAC after the operation.

### 3.3. Histological Analysis

**Figure 2:**
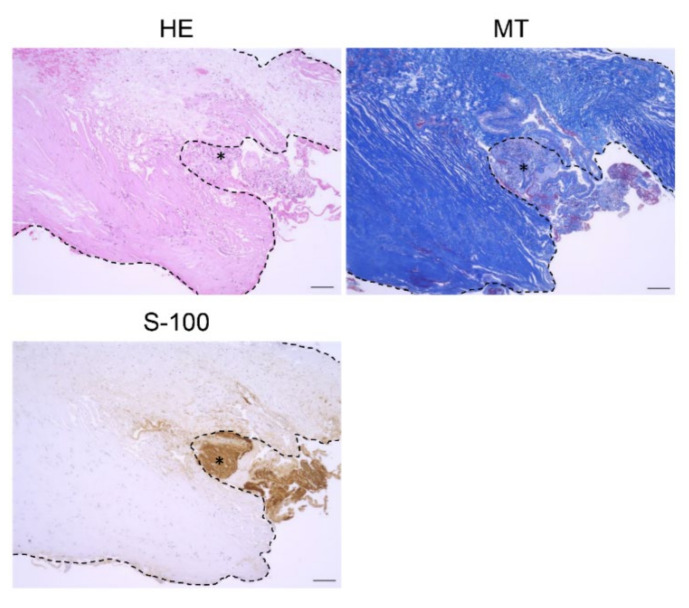
Histopathological images of DLM (dura-like membrane). DLM in Case 1 is shown. MT and S-100 stainings are shown. The thick connective tissue layer is the “DLM” that envelops the vestibular schwannoma. The tumor was positive for S-100 staining (asterisk) (scale bar= 100 μm). The dotted line represents the borderline between the tumor and DLM in each staining. HE: Hematoxylin-Eosin staining; MT: Masson’s Trichrome staining; S-100: S-100 protein immunostaining.

**Figure 3:**
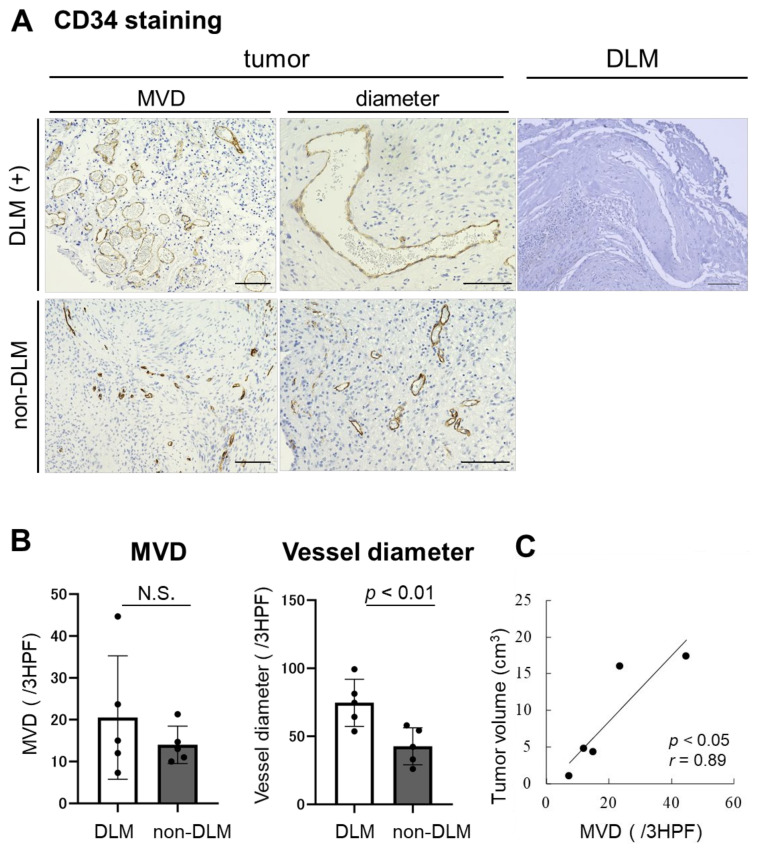
Immunohistopathological analysis of CD34 staining. (**A**) Microvessel density (MVD) and diameter analyzed using CD34 staining are shown. Large CD34(+) vessels are observed in the tumors with DLM. Few CD34(+) vessels are identified in DLM (scale bar= 100 μm). (**B**) No significant difference is observed in the MVD in the tumors between DLM group and non-DLM groups (*p* = 0.19). Vessel diameters are significantly larger in DLM group than that in non-DLM group (*p* < 0.01). (**C**) The correlation between tumor volume and MVD are shown. The larger-sized VSs demonstrate higher MVD in the tumor with DLM (*p* < 0.05, *r* = 0.89).

**Figure 4:**
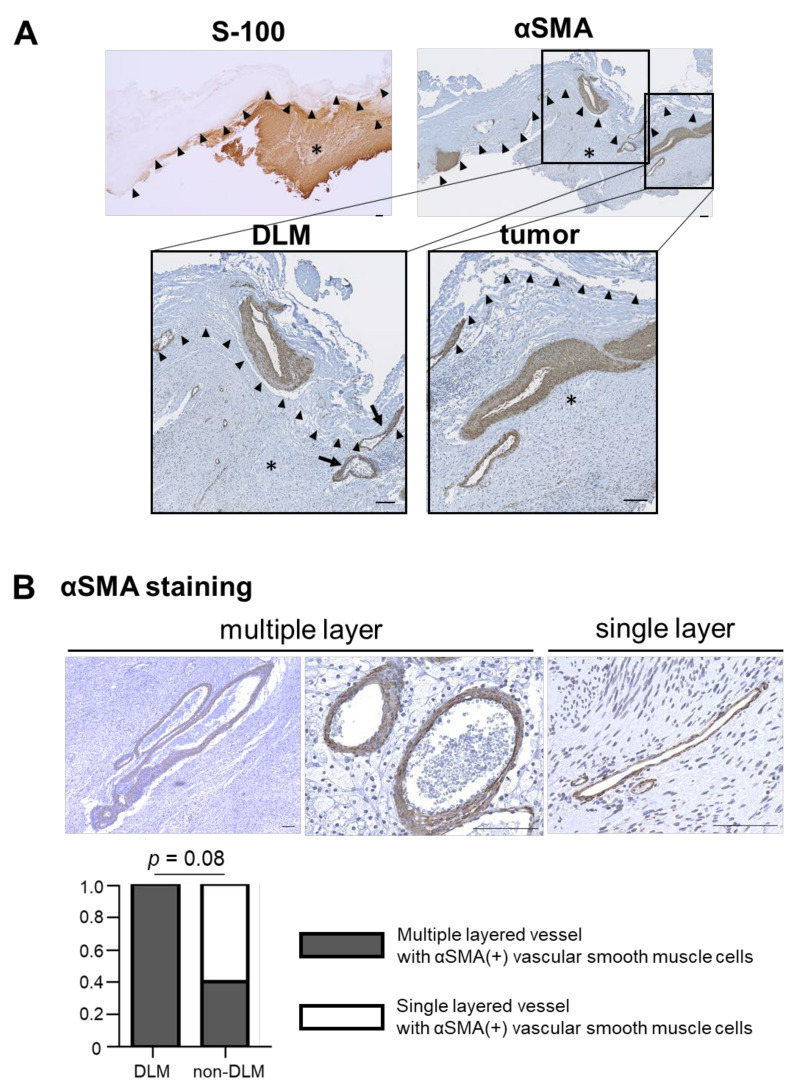
Immunohistopathological analysis of α-smooth muscle actin (αSMA) staining. (**A**) Tumor, DLM and the borderline are identified using S-100 staining. The αSMA expressions of the tumor and DLM are shown. Multilayered αSMA(+) vessels are identified in DLM, tumor, and the borderline. Low- and high-magnification images are shown (scale bar = 100 μm). Black arrow, αSMA(+) vessels located on the borderline between DLM and tumor. Black arrow head, borderline between DLM and tumor. *, tumor. (**B**) Multilayered αSMA(+) vessels tend to be more in the tumor of the DLM group than that of non-DLM group (*p* = 0.08).

**Figure 5:**
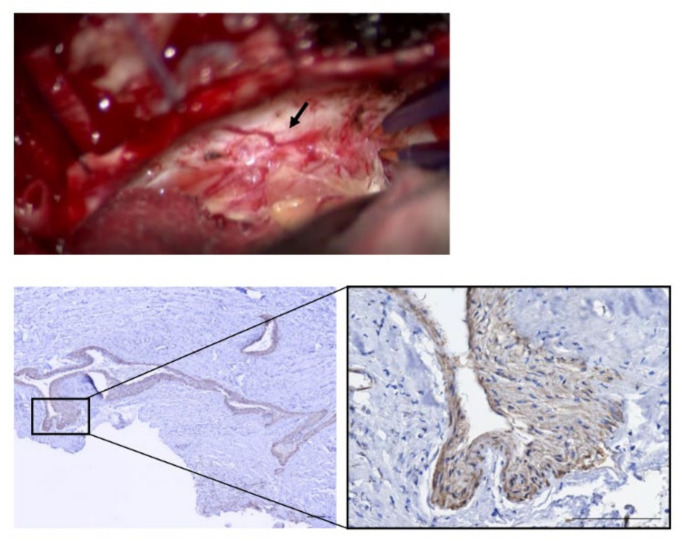
αSMA expression of the dura mater around internal auditory meatus (IAM) in Case 1. Intraoperative findings of the dura mater around IAM. The dilated vessels are shown on the dura mater (black arrow). Low- and high-magnification images are shown (scale bar = 100 μm). Large multilayered vessel with αSMA(+) vascular smooth muscle cells are observed in the dura mater, which are similar to DLM.

S-100(+) tumors were covered by a thick connective tissue layer (DLM), which stained blue with MT (Figure 2). Although there was no significant difference in MVD of CD34(+) intratumoral vessels between the DLM and non-DLM groups (20.5/3HPF vs. 14.0/3HPF, *p* = 0.19), vessel diameter was significantly larger in the DLM group than the non-DLM group (74.7 μm vs. 42.7 μm, *p* < 0.01) (Figure 3A,B). MVD of the CD34(+) vessels correlated with tumor volume in the DLM group (*p* = 0.042, *r* = 0.89) (Figure 3C). Few CD34(+) vessels were observed in the DLM (Figure 3A). In contrast, multilayered vessels with αSMA(+) vascular smooth muscle cells were observed in both the DLM and tumor in the DLM group (Figure 4A); there tended to be more of these vessels within the tumor in the DLM group compared to the non-DLM group (Figure 4B) (*p* = 0.083). Cross-boundary multilayered vessels with αSMA(+) expression were observed between the DLM and tumor (Figure 4A). The dura mater of the posterior wall of the IAM in case 1 showed multilayered vessels with αSMA(+) vascular smooth muscle cells, similar to DLMs (Figure 5).

## 4. Discussion

Schwannomas are comprised of a true capsule, which is densely attached to the tumor, and a pseudocapsule, which contains nerve tissue [11]. Our previous study demonstrated an additional DLM which sometimes envelops the VS adjacent to the IAM; recognition of this membranous structure during surgery is important in order to preserve the facial and cochlear nerves [4]. In this study, quantitative volumetric analysis demonstrated that the presence of a DLM was associated with a lower tumor resection volume.

We found that the DLM consists of thick connective tissue and multilayered vessels with αSMA(+) vascular smooth muscle cells. αSMA(+) vessels were also found in the border and within the tumor just beneath the DLM. Intraoperative findings showed that the dilated vessels proliferating on the DLM surface arose from the dura mater around the IAM. These findings resemble a feeding artery. Sitohy et al. [5] reported that tumors are supplied by six types of blood vessel which arise from both angiogenesis and arteriovenogenesis. Feeder arteries and draining veins are induced by arteriovenogenesis. The large vessels induced by arteriogenesis are coated with multiple layers of vascular smooth muscle cells [12]. Our findings suggest that tumors are fed by arteriogenesis in the DLM. As vascular smooth muscle cells produce an extracellular matrix [13], we hypothesize that they are involved in the production of the thick connective tissue of the DLM.

Tumor angiogenesis as assessed by CD31 and CD34 staining is associated with tumor growth [14]. Vascular endothelial growth factor (VEGF) upregulates a massive signaling cascade in endothelial cells, stimulating tumor angiogenesis [15]. Previous reports have demonstrated that VS growth also requires VEGF and basic fibroblast growth factor [16]. Unlike normal blood vessels, tumor blood vessels are dilated and irregularly shaped [17]. This study demonstrated that tumors with a DLM also had large and irregularly shaped CD34(+) vessels. In addition, MVD of intratumoral CD34(+) vessels correlated with tumor volume in tumors with a DLM. Angiogenesis is a vital process in tumor formation as well as the formation of collagen [7]. Activated fibroblasts promote formation of collagen [18]. Therefore, the presence of a collagen-rich DLM may be associated with high angiogenic capacity.

VSs with high arteriogenic capacity rarely induced DLM formation around the IAM in this study. The DLM consisted of thick connective tissue and multilayered vessels with αSMA(+) vascular smooth muscle cells, which may also become the source of feeding arteries. Furthermore, the formation of DLM may be associated with not only high vascularization, but also other growth factors and microenvironment described below. Walocha et al. also demonstrated the similar capsule around uterine leiomyoma. During development of leiomyoma, the pre-existing blood vessels undergo regression and new vessels invade the tumor from the periphery probably promoted by growth factors secreted by the tumor (i.e., basic fibroblast growth factor (bFGF) and adrenomedullin (ADM)). The formation of a ‘vascular capsule’ was responsible for supply of blood to the growing tumor under the hypoxic condition [19].

The main limitation of the present study was the small number of VSs with DLM. Studies using a larger number of patients are warranted to confirm our findings. In addition, only VSs were analyzed; a study examining schwannomas arising from other cranial nerves should be conducted to generalize our results. Although the presence of the DLM has a possibility to negatively affect the surgical outcome in some cases, the resection rate and functional preservation of the facial and cochlear nerves may be associated with several factors including tumor volume, preoperative hearing status, age, and expertise of the surgeons. Further multivariate statistical analysis is needed.

## 5. Conclusions

The existence of DLM was associated with difficult and lower volume of VS resection. VSs with a DLM were demonstrated to have strong arterio- and angiogenic characteristics. Multilayered vessels with αSMA expression were observed in the border between the DLM and tumor. These characteristics suggest that the DLM is formed as the tumor induces feeding vessels from the dura mater around the IAM.

## Figures and Tables

**Table 1:** Patients’ characteristics.

Case	Age	Sex	DLM	Preoperative Symptom	Postoperative Symptom	Tumor Volume (cm^3^)	Resection Rate (%)	Rec
1	35	M	+	hearing disorder (G-R Gr.3) facial pain	facial n. palsy (H-B Gr.4, permanent)	16	100	-
2	45	M	+	hearing disorder (G-R Gr.2) gait disturbance hydrocephalus	no change	17.4	70	-
3	21	F	+	hearing disorder (deaf)	no change	4.8	90	-
4	61	F	+	hearing disorder (G-R Gr.2)	no change	4.4	82	-
5	67	F	+	hearing disorder (G-R Gr.3)	hearing disorder (deaf)	1.1	64	-
6	66	M	-	hearing disorder (G-R Gr.3)	facial n. palsy (H-B Gr.3, temporary)	0.3	100	-
7	54	M	-	hearing disorder (G-R Gr.3)	no change	1.3	92	-
8	35	F	-	hearing disorder (G-R Gr.3)	no change	0.9	100	-
9	50	M	-	hearing disorder (G-R Gr.2) facial numbness	hearing disorder (deaf) facial n. palsy (H-B Gr.4, temporary)	4.3	100	-
10	60	F	-	hearing disorder (G-R Gr.4) facial n. palsy (H-B Gr.2) facial numbness	no change	16.6	100	-

DLM, dura-like membrane; F, female; G-R Gr, Gardner–Robertson Grading; H-B Gr, House–Blackman Grading; M, male; N, nerve; Rec, recurrence.

## Data Availability

The datasets generated and/or analyzed during the current study are available from the corresponding author upon reasonable request.
